# Can Mean Platelet Volume and Platelet Distrubition Width be Possible Markers for Ectopic Pregnancy and Tubal Rupture? (MPV and PDW in Ectopic Pregnancy)

**DOI:** 10.12669/pjms.302.4177

**Published:** 2014

**Authors:** Burcu Artunc Ulkumen, Halil Gursoy Pala, Esat Calik, Semra Oruc Koltan

**Affiliations:** 1 Burcu Artunc Ulkumen, Celal Bayar University School of Medicine, Obstetrics and Gynecology Department, Manisa, Turkey.; 2 Halil Gursoy Pala, Celal Bayar University School of Medicine, Obstetrics and Gynecology Department, Manisa, Turkey.; 3 Esat Calik, Celal Bayar University School of Medicine, Obstetrics and Gynecology Department, Manisa, Turkey.; 4 Semra Oruc Koltan, Celal Bayar University School of Medicine, Obstetrics and Gynecology Department, Manisa, Turkey.

**Keywords:** Ectopic pregnancy, Mean platelet value, Platelet distribution width, Platelet count

## Abstract

***Objective:*** We aimed to evaluate the alterations in serum levels of platelet indices such as mean platelet volume (MPV) and platelet distribution width (PDW) in ectopic pregnancy (EP) and discuss the mechanism of the alterations in MPV and PDW.

***Methods:*** This retrospective evaluation of 153 tubal EP patients (39 ruptured and 114 non-ruptured) admitted to our clinic between 2009 and 2013 and 67 healthy pregnancies was conducted. The data regarding the maternal age, hemoglobin level, platelet level, MPV, PDW was analyzed.

***Results:*** MPV was lower in the EP, especially in ruptured EP, compared to control group. However, no significant difference could be found between the groups (p=0.616). PDW was higher in the EP, especially in ruptured EP, compared to control group, however there was no statistical difference between the three groups (p=0.451). Platelet counts were significantly lower in ruptured EP compared to non-ruptured ectopic pregnancies and control groups (p=0.005).

***Conclusions: ***MPV seems to be lower in ruptured EP suggesting the possible high grade inflammation in pathology. Platelet counts tend to be lower in ruptured EP suggesting the consumption of the platelets at the inflammation site. However, further studies are needed to describe the usefulness of the platelet indices in the diagnosis and clinical follow-up of EP. Our preliminary results show that MPV levels may decrease in the ruptured EP cases. At the same time, PDW levels may increase.

## INTRODUCTION

Ectopic pregnancy (EP) is a clinical condition in which the embryo implants outside the uterine cavity. Approximately 1-2% of all pregnancies result in ectopic implantation.^1^ Most ectopic pregnancies occur in the fallopian tubes which are known as ‘tubal ectopic pregnancy’ (TEP). The main risk factor for TEP is tubal damage mostly due to tubal surgery –including tubal ligation-, previous ectopic pregnancy and assisted reproductive technologies.^2^ The pathogenesis depends on the defective tubal transport due to the tubal ciliary dysfunction and the changes in the micro environment of the fallopian tube leading early implantation of the fertilized ovum.^1^

Recent studies have highlighted the role of platelets and platelet-derived agents in thrombosis, angiogenesis, inflammation and immunity.^3^ In ectopic pregnancy, some inflammatory cytokines are increased both at the implantation site and systemic circulation.^1^

We aimed to evaluate the alterations in platelet indices like Mean Platelet Volume (MPV) and Platelet Distribution Width (PDW) in the inflammatory status of ectopic pregnancy. As far as we know, the alterations in the MPV and PDW levels in EP was not evaluated before. The only study about MPV and leukocyte counts showed an increase in their levels.^4^ The aim of our study was to evaluate the alterations in platelet indices like MPV and PDW in the inflammatory status of ectopic pregnancy and discuss the mechanism of the alterations in MPV and PDW.

## METHODS

This retrospective study was carried out at the department of obstetrics and gynecology of a tertiary center and was approved by the Institutional Ethics Committee. The data of 153 TEP patients (39 ruptured and 114 non-ruptured) between 2009 and 2013 was analyzed. The control group consisted of 67 healthy pregnant women during their first trimester. The data regarding the maternal age, hemoglobin level, platelet level, mean platelet volume (MPV), platelet distribution width (PDW) were evaluated. Patients with chronic inflammatory diseases such as connective tissue disorders such as systemic lupus erythematosus, rheumatoid arthritis, vasculitis, renal or hepatic insufficiency, hemoglobinopathies, diabetes mellitus, hypertensive disorders, previous myocardial infarction and previous thrombosis history were excluded from the study. Hemodynamically stable TEP were included in the study.

The blood samples were taken just after the admission to our clinic. All blood samples were collected in tubes with EDTA (potassium ethylenediaminetetraacetate) which served as the anticoagulant agent. All the blood samples were analyzed by the hematology analyzer in two hours (MINDRAY BC-6800).

The data was analyzed using the Statistical Package for Social Sciences (SPSS) software version 20. A two-tailed p value <0.05 was regarded as statistically significant for all comparisons. T-test was used to compare to different groups and One-way ANOVA test was used to evaluate the intergroup differences.

## RESULTS

The mean age of TEP was 29.55.8 and the mean age of the control group was 27.55.5. There was no significant difference regarding the mean age of the groups (p=0.515). Platelet values, mean platelet volume and platelet distribution width levels of the groups (TEP-ruptured and non-ruptured- and controls) are shown in Table-I. The mean hemoglobin levels were 12.12.3 g/dl and 12.33.1 g/dl in TEP and control group respectively (p 0.918). The mean platelet levels were 234.17064.530 /mm^3 ^and 228.49063.810 /mm^3^, MPV levels were 9.215.8 fL and 9.731.03 fL, PDW levels were 16.230.75 and 16.110.57 in TEP and in control group respectively. Regarding the platelet functions, there was no statistically significant difference between the TEP and control groups. Between the subgroups -ruptured and unruptured TEP-, there was also no difference (p=0.616, p=0.451 regarding MPV and PDW respectively). However, in ruptured TEP group, the mean platelet levels were significantly lower than the unruptured TEP and control groups (p<0.005) (Table-II).

## DISCUSSION

Ectopic pregnancy occurs due to the combination of defective tubal transport of the fertilized ovum and the changes in the microenvironment of the tubal lumen leading to the early implantation before entering the uterine cavity.^1^ TEP is one of the significant causes of maternal mortality, with an incidence of 0.4 per 1000 ectopic pregnancies in UK, standing for four maternal deaths, annually.^5^

**Table-I:** Platelet indices in TEP and control group

	*Ectopic pregnancy * *(n=153)*	*Control group* *(n=67)*	*p*
Mean age	29.495.8	27.465.5	0.170
Hb (g/dl)	12.152.3	12.363.1	0.630
Platelet (/mm^3^)	234.17064.53	228.49063.81	0.546
MPV	9.215.8	9.731.0	0.285
PDW	16.230.75	16.110.57	0.201

**Table-II:** Platelet indices in ruptured and nonruptured TEP and control group

	*Ruptured TEP (n=39)*	*Non-ruptured TEP (n=114)*	*Control group* *(n=67)*	*p*
Mean age	29.844.6	29.376.2	27.465.5	0.052
Hb (g/dl)	11.721.58	12.32.8	12.363.2	0.426
Platelet (/mm^3^)	206.0852.8	243.7865.6	228.4963.8	*0.005*
MPV	8.761.0	9.366.7	9.731.0	0.616
PDW	16.280.8	16.220.7	16.120.6	0.451

The diagnosis of EP is sometimes distracting due to the various clinical presentations of the patients: some cases may be asymptomatic, some have acute abdomen, whereas some have hemodynamic shock.^6^ In general, 1/3 of patients are asymptomatic and 9% of asymptomatic cases present with rupture.^5^ The diagnosis is mainly based on the combination of the ultrasonography findings and serum beta human chornonic gonadotropin (β-HCG) levels. With a single β-hCG level -especially less than 1500 mİU/mL, the differential diagnosis between ectopic pregnancy (EP) abortion and early intrauterine pregnancy is troublesome at first examination.^7^ At least 66% increase in β-hCG serum level is suggestive for a healthy intrauterine pregnancy. As during the follow-up period these patients will stay at hospital, the diagnostic process with serial serum -hCG sampling and follow-up with sonography will be time-consuming.^6^ Therefore, recent researches pointed to a biomarker which could distinguish an ectopic pregnancy at first examination.^7^

Complete blood count (CBC) is routinely checked for all pregnant women. Platelet count and platelet indices such as MPV and PDW are parameters of the routinely checked CBC. As the platelets are natural sources of growth factors like platelet-derived growth factor (PDGF), vascular endothelial growth factor (VEGF), insulin-like growth factor 1 (IGF-1) or transforming growth factor β (TGF-β), they have important role in inflammation, angiogenesis, repair and regeneration of the tissues.^7^ The activation of the platelets causes some morphological alterations: the activated platelets seem larger by becoming spherical in shape and forming pseudopodia. As a result, platelets with enhanced number and size of pseudopodia will be different in size leading alterations in PDW.^8^ In tubal ectopic pregnancies –either ruptured or not-, due to inflammation and changes in the microenvironment of the fallopian tube, platelet activation indices like MPV.^4^ and PDW should be altered than the controls. Previous studies suggested that MPV levels decreased in low dose inflammation processes, whereas increased MPV values were found in high level inflammatory disorders.^9^

All blood samples in our study were collected in tubes containing potassium ethylenediaminetetraacetate (EDTA) as the anticoagulant agent and they were analyzed in two hours following the sampling. However, during blood storage, MPV and PDW levels change in a time-dependent manner. Vagdatli et al. showed an increase in MPV over time and a decrease in PDW levels.^8^ The time period between the blood sampling and sample analyzing affects the levels of platelet indices leading to unreliable results.^4^ In emergent conditions such as in ectopic pregnancies, the blood samples are analyzed immediately. However, the samples of the control group are analyzed in two hours which is enough time for MPV levels to increase and for PDW levels to decrease. So, the comparison of platelet indices between ruptured and non-ruptured tubal pregnancies seem to be more reasonable. Furthermore, the use of different anticoagulant agents in the blood tube also affect the platelet indices.^8^

In our study, we found that MPV was lower in EP patients when compared to controls. Furthermore, EP patients with tubal rupture had lower MPV values than unruptured EP cases. In contrast, PDW levels were higher in ruptured EP patients compared to controls. To the best of our knowledge, this is the first study evaluating the PDW levels in EP. However, the change in MPV levels was studied before by Turgut et al. In contrast to our results, Turgut et al. showed that EP patients had significantly higher MPV levels.^4^ Platelet activation causes MPV levels to increase. However, platelet indices like MPV and PDW are not only increased due to platelet activation, also hemorrhage can lead some increment in platelet size and volumes.^8^ A direct association between MPV and platelet activation is not easy to establish; because, the reliability of the results in the previously studies depend on the preanalytical factors; such as the time between sampling and analyzing, the anticoagulant agent in the tube.^9^^-^^11^ Platelet indices may vary also depending on the gestational week. In general, dilutional thrombocytopenia exists with an compensatory increase in MPV and PDW levels during the pregnancy.^10^ Cardiovascular risk factors like smoking status, hypertension, dyslipidemia, diabetes also affect the size of platelets.^12^ Apart from this, in inflammation via some cytokines, the platelets size and volume alter differently: in low grade inflammatory disorders, by the involvement of the large platelets in thrombi, MPV values may increase. However, in high grade inflammatory conditions, the consumption of the large platelets at the inflammation site cause a decrease in MPV levels.^9^ In TEP, –especially in ruptured cases- a high grade inflammation occurs at the implantation site. Higher degree of inflammation occurs at the rupture site of the fallopian tube. Lower MPV levels in our study –which is different from the previous data- may be the result of this high inflammation. Furthermore, increasing values of PDW levels in EP may favor the platelet activation in the pathogenesis together with this high inflammation.

As a result, our preliminary findings show that MPV levels may decrease in the ruptured EP cases. At the same time, PDW levels may increase. However, only one blood sample would not be efficient. We suggest that a decreasing trend in MPV levels and an increasing level in PDW levels in serial measurements may favor ruptured TEP which must be further investigated.

## Authors Contribution:

1. Burcu Artunc Ulkumen conceived, designed the study, did statistical analysis & writing of manuscript

2. Halil Gursoy Pala: Analysis of the data, writing and editing the manuscript.

3. Esat Calik did data collection.

4. Semra Oruc Koltan did review and final approval of the manuscript 
